# The Mouthwash

**Published:** 1907-06-29

**Authors:** 


### Dental Department.

### THE MOUTHWASH.

### Some Points in the Hygiene of the Teeth.

A few minutes' attention to the natural conditions prevailing in the oral cavity will make it clear how neglect of simple precautionary measures here may lead to general constitutional disturbances affecting the health of the individual. Pure saliva, slightly alkaline in reaction as it is, possesses practically no antiseptic properties, and consequently the oral cavity is a happy growing ground for bacteria. The variety of micro-organisms which have been found dwelling upon the mucous membrane of the mouth is indeed astonishing. They range from the humble and comparatively harmless Streptococcus albus to the bacilli of pneumonia and diphtheria, and it is safe to assert that if a bacteriological examination was made regularly no mouth would be found free from one or more pathogenic organisms. The interstices between the teeth, and between them and the gums are ideal places for the lodgment of micro-organisms. When artificial teeth are worn, without proper attention being paid to the systematic and regular cleaning of the dentures, additional lodging-places are provided. The natural protective barrier against bactericidal infection lies behind the teeth, in the fauces. Here the ring of lymphatic tissue, the lingual, pharyngeal, and faucial tonsils normally form a first defence against invasion. They are constantly dealing with organisms, and usually they are effective enough in preventing invasion. But when additional strain is thrown upon them, when decomposing material is allowed to remain between the teeth or the dentures, and when the mouth is badly cleansed or not cleansed at all, their efficiency is seriously impaired. Under such conditions, when the vitality of the organism is decreased by illness or other causes, the barrier is passed by the bacteria which flourish in the mouth, and septic absorption takes place.

The care of the teeth is a matter which is too often left entirely in the hands of the dentist, whereas the family practitioner, by judiciously pointing out the proper means of safeguarding the patient from infection through the mouth, is often in a position to forestall the necessity of a visit to the dentist. Where the patient is suffering from a serious illness, such as enteric or pneumonia, or where he has undergone an operation, the practitioner will naturally have to give his attention to the care of the mouth, and the question of prescribing a suitable mouthwash will require consideration.

There are at the present moment a number of registered preparations on the market, all of which claim to be efficient mouthwashes or dentifrices, and most of which are eminently suitable as such. It is probable that all of them are very efficient antiseptics. The volatile oils which they contain and the direct antiseptic constituents which enter into their composition, make them so. Most of them, however, are comparatively expensive, and the practitioner can easily substitute as efficient and as pleasant a mouthwash as any on the market by prescribing a cheap antiseptic combined with some appropriate flavouring. In acute specific fevers, where a strong mouthwash is usually inadvisable, the best combination is generally a few grains of sodium bicarbonate, mixed with an equal quantity of sodium biborate, and dissolved in an ounce of distilled water to which a drachm of glycerine and a minim or two of tincture of myrrh have been added. The addition of the glycerine renders the mouthwash more efficient by keeping the mucous membrane wet, and at the same time its sweetish taste stimulates the oral mucosa to a slight extent. In cases of scarlet fever or typhoid, where a stronger mouthwash may be desirable, chlorate of potash solution is as good as any. A chlorate of potash mouthwash, indeed, is one of the best for smokers' use, and is one of the most reliable oral antiseptics.

Where artificial teeth are worn the use of a brush alone will not ensure the cleaning of the mouth. An ordinary soft hair brush will not penetrate between the crevices of the natural teeth; the indiarubber brushes still on the market are useless, since they only brush the surface of the gums, and cannot be made to cleanse the spaces between the teeth. The old-fashioned silk thread, soaked in a mouthwash before use, remains one of the most perfect of tooth cleansers, but its use is irksome, sometimes painful, and by no means economical, so far as time is concerned. The patient with artificial teeth or a denture should be made to clean what natural teeth he possesses as efficiently as he is able with a soft, long-bristled tooth-brush, using the mouthwash or dentifrice freely. The denture should be put in the mouthwash itself, and should be carefully cleansed with a special brush.

One of the most pleasant and, from a point of view of antisepsis, one of the strongest mouthwashes is that in which the base is methyl salicylate, or one of the carbolic acid group. A mixture such as the following is exceedingly pleasant to use, and can easily be made up at a trifling cost:—

| | | |
|---|---|---|
| ℞ | Tinct. Quillaiæ ... ... ... ... | ℥j. |
| | Methyl salicylatis ... ... ... | ♏xxx. |
| | Ol. bergamot ... ... ... ... | ♏xx. |
| | Ol. thyme ... ... ... ... ... | ♏x. |
| | Ol. geranii ... ... ... ... ... | ♏xij. |
| | Vanillin ... ... ... ... ... | gr. iij. |
| | Saccharin ... ... ... ... ... | gr. iij |
| | Spirit. vini rect. ad ... ... ... | ℥iij. |
| M. fiat dentifricium. | | |

This is a clear, straw-coloured solution. A few drops of it are put into a tumblerful of water, preferably lukewarm, which it renders opaque owing to the precipitation of the essential oils which are held in solution by the tincture of quillaia. This opalescent solution makes an efficient mouthwash for most cases where one is required. The denture should be soaked in this for a few minutes, while the mouth is rinsed and the teeth brushed with a similar solution.

